# Effects of Infill Plate’s Interconnection and Boundary Element Stiffness on Steel Plate Shear Walls’ Seismic Performance

**DOI:** 10.3390/ma15165487

**Published:** 2022-08-10

**Authors:** Nima Paslar, Alireza Farzampour

**Affiliations:** 1 Department of Civil Engineering, Islamic Azad University of Qeshm, Dargahan 0782-362, Iran; 2 Department of Civil Engineering, Payame Noor University (PNU), Tehran P.O Box 19395-4697, Iran; 3 Department of Permitting, Inspections and Enforcement, Washington, DC 20774, USA

**Keywords:** steel plate shear walls, connection, boundary elements, stiffness

## Abstract

Steel plate shear walls (SPSWs) are among the most desirable load-bearing systems, which have been used wildly in various structures. Recently, designers have tended to SPSWs with only beam connections showing several problems. In the present research, several SPSWs with various types of connection conditions between infill plate and boundary elements, and various stiffness of boundary elements have been studied. The result illustrates that by having the full connection between infill plate and boundary elements, at least a 33% interconnected infill plate to columns could eliminate the significant loss of fundamental factors in SPSWs connected to beam only. Furthermore, increasing the stiffness of columns has more effect on the performance of SPSWs in comparison with beams.

## 1. Introduction

SPSWs are used in many various lateral-load-resisting systems for having low weight, fast and appropriate workability, significant ultimate strength, sufficient ductility, and stiffness. Previous studies indicated the advantages of the use of these systems in significantly space-constrained areas and high seismic conditions [1,2,3]. Recent investigations allocated to improve the performance of this system, by limiting the interconnection interactions between the plate and the boundary elements [4,5], temperature field reconstruction [6], stiffened SPSWs [7,8], various geometrical shapes and infill plate conditions [9], effects of openings [10,11], infill plate types such as corrugated infill plates [12,13,14], and interconnection ratio [15,16], were studied to overcome previously reported structural issues such as excessive demands on columns, detrimental impacts of openings on SPSWs, problems in the buckling of infill plates, and so on.

The cantilever wall system of SPSWs is developed, consisting of a vertical steel plate connected to surrounding boundary element members [15,16,17,18,19,20,21,22,23,24,25,26,27,28,29,30,31]. Several previous studies signified the high stiffness, desirable energy-dissipation capability, efficient use of the space, and adequate ductility in SPSW applications [32,33,34,35]. The use of SPSWs in structures with significant importance (e.g., Olive View hospital), in high-rise buildings for providing an efficient lateral-load-resisting system (e.g., a 35-story building in Kobe, Japan), and in residential applications (e.g., LA Live Hotel), indicated efficient space usage, lower demand forces to the boundary, desirable performance under earthquakes, reduction in the total weight of structures, and convenient construction procedures [36,37].

Under lateral cyclic loading conditions, the SPSW undergoes in-plane and out-of-plane deformations. Due to the slenderness of the infill plate, buckling occurs at the initial loading. The infill plate folds in an out-of-plane direction, initiating the diagonal tension field action parallel to the principal tensile stresses, which leads to the postbuckling resistance capacity. Several studies indicated that the postbuckling strength of these walls is highly dependent on the development of tension field action, which is completely affected by the infill plate’s interconnectivity with boundary elements [38,39]. Several procedures have been developed to reduce the demand forces on the boundary elements, which necessitates that the infill plate could be detached from the columns. The detachment of the infill steel plate leads to fewer demand forces generated by tension field action on the columns; hence, smaller boundary element sections are required [40,41,42].

From the laboratory investigations, it was shown that those SPSWs that infill plates were only attached to the one of the boundary elements; several desirable performances were obtained [42,43,44,45,46]. The ultimate results indicated that in the initial stages of the loading, the infill plate would undergo significant dissipation of the energy; however, by increasing the loading amplitude, the yielding inside of the plate started to spread over the infill plate, and subsequently to the frame boundary members [47]. In addition, it has been reported that a large number of shear forces were resisted by boundary columns. The upper bound value recorded for the drift in each story was quite smaller than the allowable value of 2.5%, suggested by NBCC 2015 (National Building Code of Canada 201) [43,44].

Although only some research has been conducted regarding the performance of SPSWs with connection to beams, there are some hidden points about the minimum desirable interconnection between the infill plate and boundary elements and the simultaneous effects of stiffness of boundary elements on the performance of SPSWs. In this study, a number of computational SPSW models with various types of connection conditions of the infill plate to boundary elements and boundary element stiffness were designed based on the verified experimental specimen of Choi et al. [48]. All models were investigated and compared to achieve a reliable optimized type of connection between the boundary elements and infill plate. In addition, the effect of increasing the stiffness of each element on the performance of the lateral resisting system has been investigated separately.

## 2. Materials and Methods

**Figure 1:**
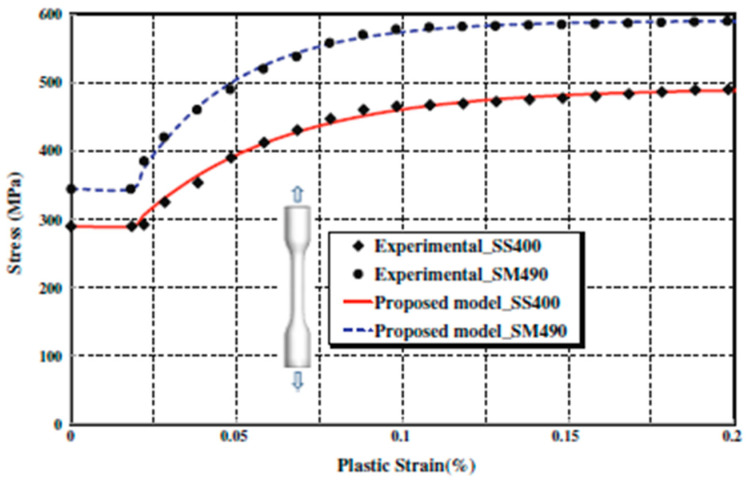
Stress–strain curves of SS400 and SM490 [50].

In the present research, a parametric study, according to previous research methods [4,49], based on the changes in the type of interconnections between infill plate and boundary elements and increasing the stiffness of boundary elements, was conducted. Experimental specimen of Choi et al. [48] was utilized for validation of finite element (FE) models, then a number of models were simulated with the aforementioned changes. Finally, all models were compared to investigate the effect of changes in ultimate strength, energy absorption, and stiffness of the models. The width and height of the specimen were 2500 mm and 3550 mm, respectively. Infill plate was made of SS400 steel (Fy = 299 MPa, Fu = 372 MPa) with 4 mm thickness, and boundary elements were made of SM490 (Fy = 377 MPa, Fu = 527 MPa for beams) (Fy = 348 MPa, Fy = 522 MPa for columns). Stress–strain curves of steel are shown in Figure 1**.** Columns were chosen to be H-150 × 150 × 8 × 20; beams of the first and second floors’ boundary elements were H-150 × 100 × 12 × 20; and the beam of the third floor was H-250 × 150 × 12 × 20. Loading was applied to the top beam of the specimen under ATC-24 protocol.

### 2.1. Verification

**Figure 2:**
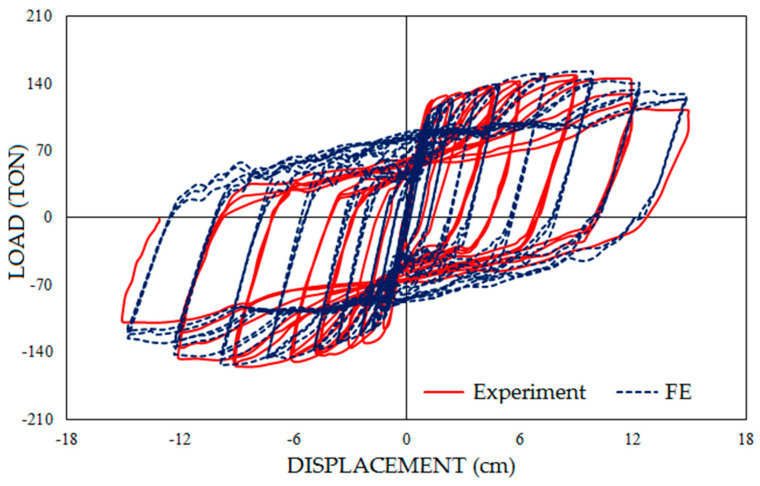
Comparison between hysteresis graphs of the experimental specimen and FE model.

**Figure 3:**
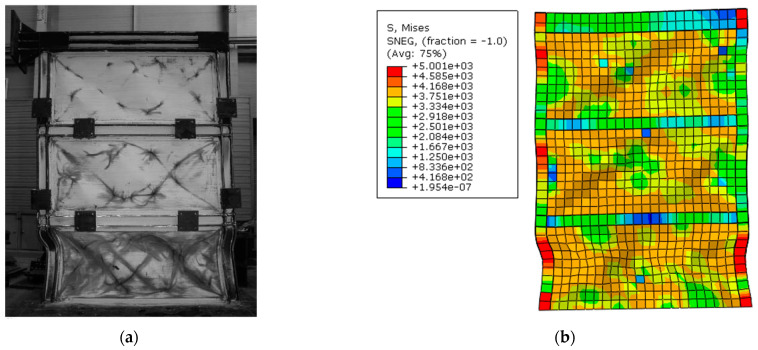
Experimental and finite element model after loading. (**a**) Experimental model [33]. (**b**) Finite element model.

The verification specimen was modeled according to the experimental specimen’s geometry and material by ABAQUS 6.14-2. S4R elements were utilized for the FE model and the mesh size was 100 mm. Boundary conditions were defined in a way in which supports were rigid and out-of-plane movements of the specimen in the location of the beam and column connection were prevented. Hysteresis graphs of the FE model and experimental model are shown in Figure 2. Furthermore, the FE model and experimental model after loading are represented in Figure 3, which shows more than 96% accuracy in FE modeling.

**Figure 4:**
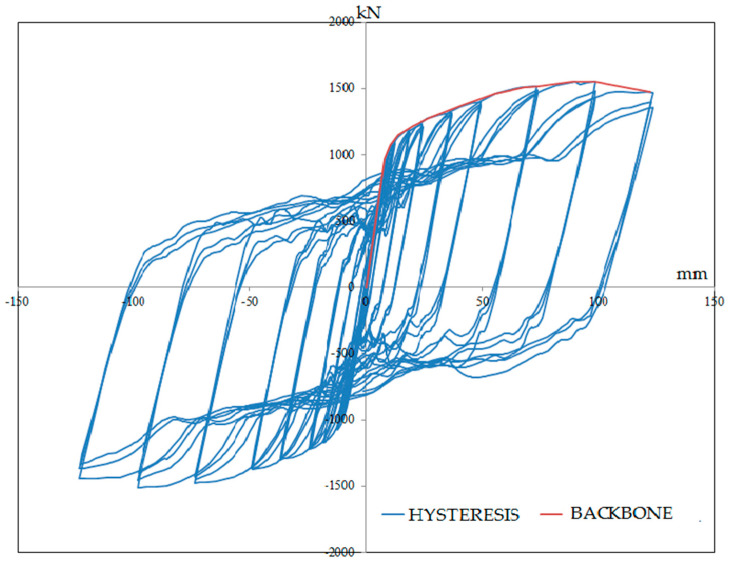
Hysteresis and extracted backbone graph of FE model.

Comparison between the FE model and experimental specimens are shown in Figure 3a,b which illustrates the accuracy of FE modeling. Columns in the FE model were buckled similar to the experimental model due to the soft story on the first floor. From the geometry properties, it can be observed that beam elements were stronger than columns and it can refer to the soft story on the first floor; furthermore, the shape of tension field actions of the infill plates were the same. In addition, for postprocessing and further investigations, the backbone curves should be obtained. Figure 4 shows the backbone graph of the S1 model that was extracted through the hysteresis graph.

### 2.2. Description of Models

**Figure 5:**
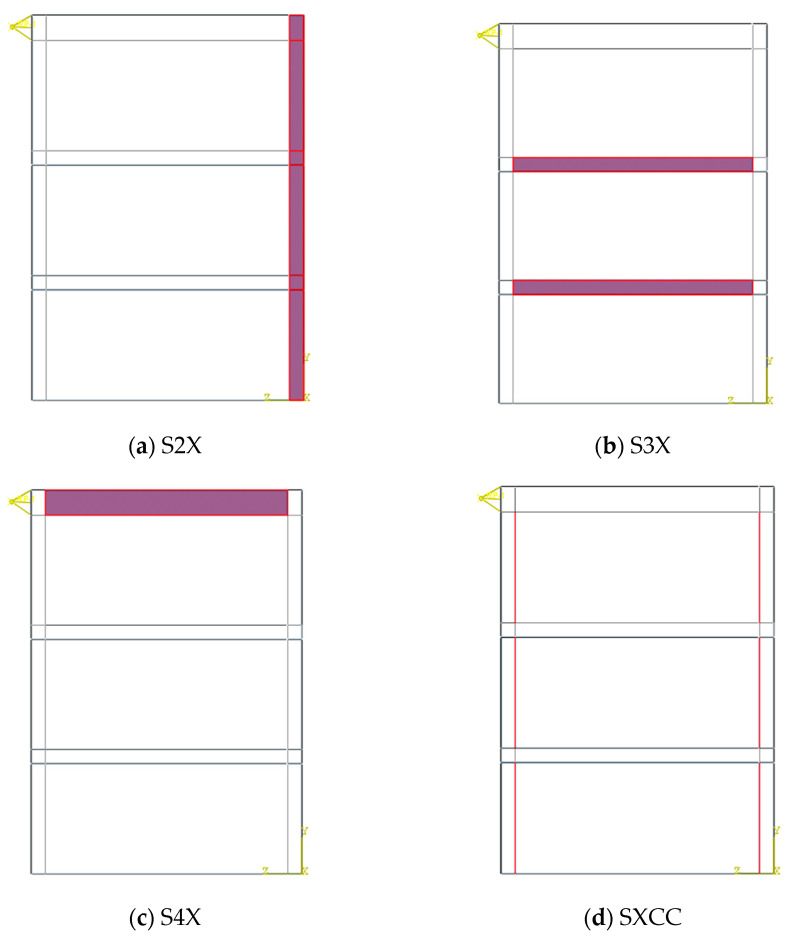

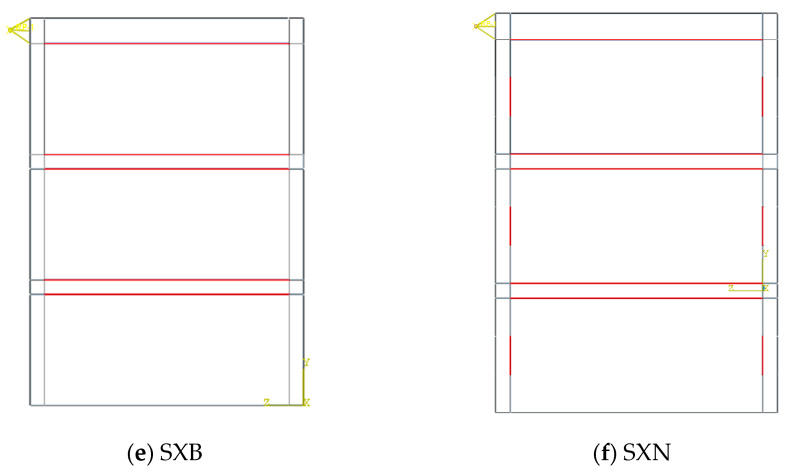
Changes in the boundary elements and connections of the FE models.

All FE models were designed by modifying the verified computational model (S1). The geometry and materials of all models were similar to the S1 model; however, they had some changes in other sections, such as stiffness of elements and the type of connection between infill plate and boundary elements. Stiffness of the one of the columns in some models was increased by 100%; these were called S2X models. S3X models had a 100% increase on the beams of the first and second floors. S4X models experienced a 100% increase on the beam of the third floor. Moreover, SXC and SXB models were designed in a way that infill plates had connections just with columns or beams. In the former model, the infill plate has connections only with the columns and the latter has connections with beams. Another type of connection is displayed in the SXN models. These models have full connection of infill plate with beams and 33% with columns. The changes in designed models are shown in Figure 5. Table 1 indicates the description of the models studied and the structural details. Table 1 presents the name of models and changes in boundary elements that were designed by changes in S1 model.

## 3. Results and Discussion

**Figure 6:**
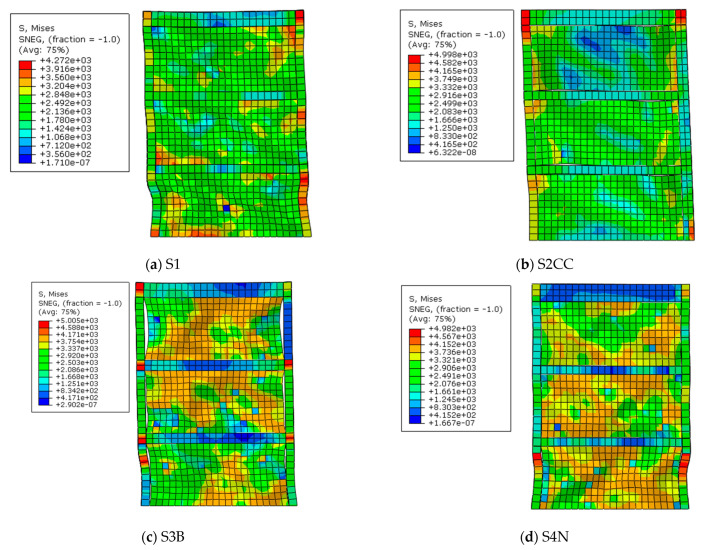
FE models under loading.

In the present section, some of the FE models after loading were investigated and shown in Figure 6**.** In all models, columns buckled on the first floor, which relates to the soft story formation on this floor due to higher stiffness of the boundary elements, and beams in particular. From Figure 6a, infill plates had full connection to boundary elements and the infill plates yielded. Figure 6b shows the capacity of the infill plates that have not been used, entirely because of the absence of connection between the infill plates and beams. Hence, it was predictable that beams were under low pressure in comparison with other types of connections in which they experienced lower stresses. Furthermore, increasing the stiffness in the right column of the models is the most important factor for having less buckling formation in the columns.

Figure 6c shows that the infill plate of the S3B model yielded, which was due to appropriate tension field action. Plastic hinges were observed in the beams of the first and second floors. The issue can be referred to the connection of the infill plate only to the beams. Increases in stiffness on the first and the second beams imposed more stress on columns and beams under the column connection zone. Figure 6d illustrates the formation of tension fields that occurred in the infill plates of the S4N model owing to having more connection in comparison with the S3B model. This type of connection between the infill plate and boundary elements can be used instead of the classic type of connection (full connection between infill plate and boundary elements), leading to a reduction in the stress on the beam to the column zone, and better performance in comparison with the models that have connections between the infill plate and beam only.

**Figure 7:**
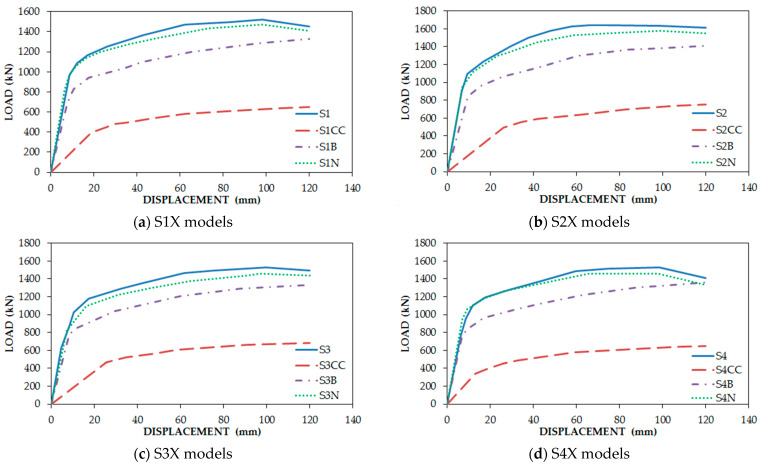
Comparison among backbone graphs of FE models based on the type of connections.

**Figure 8:**
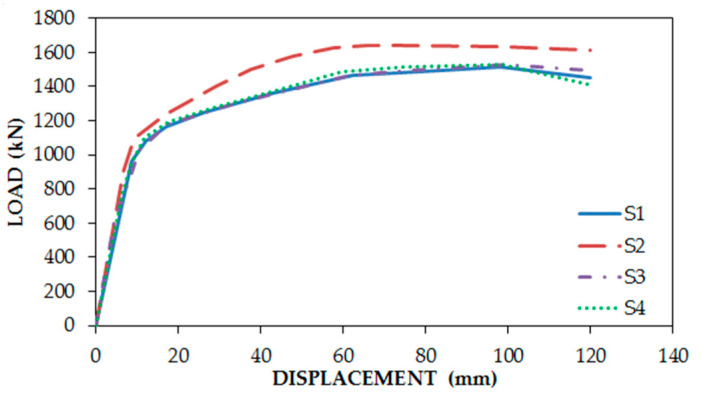
Comparison among backbone graphs of FE models based on the stiffness of the boundary elements.

Figure 7 demonstrates that the S (number) models (full connection between the infill plate and boundary elements S1, S2, S3, and S4) had the best response, and SXN (33% connection of the infill plate to columns and full connection to beams) followed them in the second rank. In addition, it can be inferred that SXCC models (infill plate connection to columns only) experienced a dramatic fall in fundamental factors of structures. Although SXB models (Infill plate connection to beams only) had better performance in comparison with SXC models, they had a considerable loss in comparison with the classic type of connection. Figure 8 solely indicates increasing the stiffness of the beams, having a neglectable effect on fundamental factors, in particular ultimate strength, but the increase in only one column had considerable effect. Table 2 illustrates the result of fundamental factors for models.

**Figure 9:**
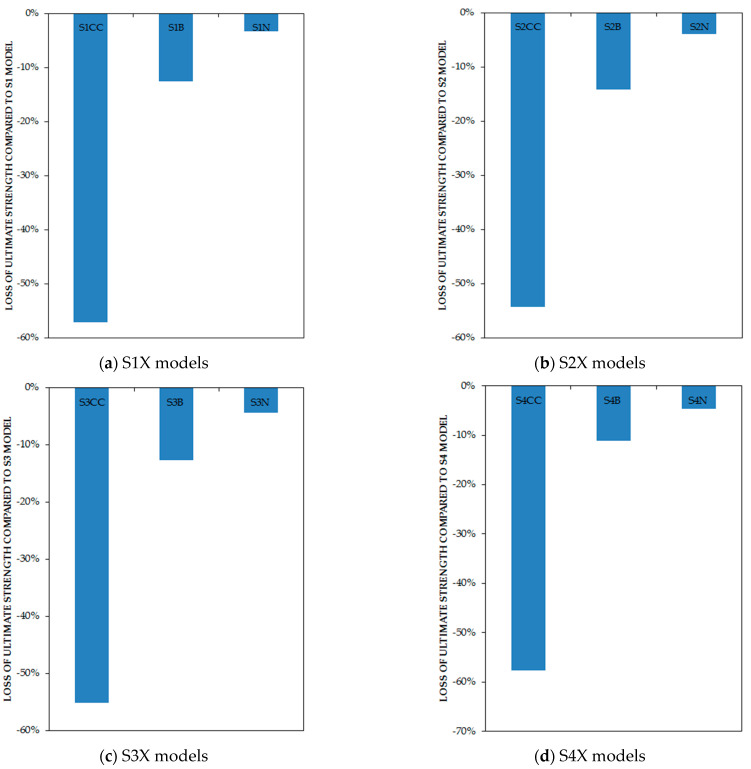
Loss of ultimate strength of models based on the type of connections in comparison with full-connection models by percentage.

**Figure 10:**
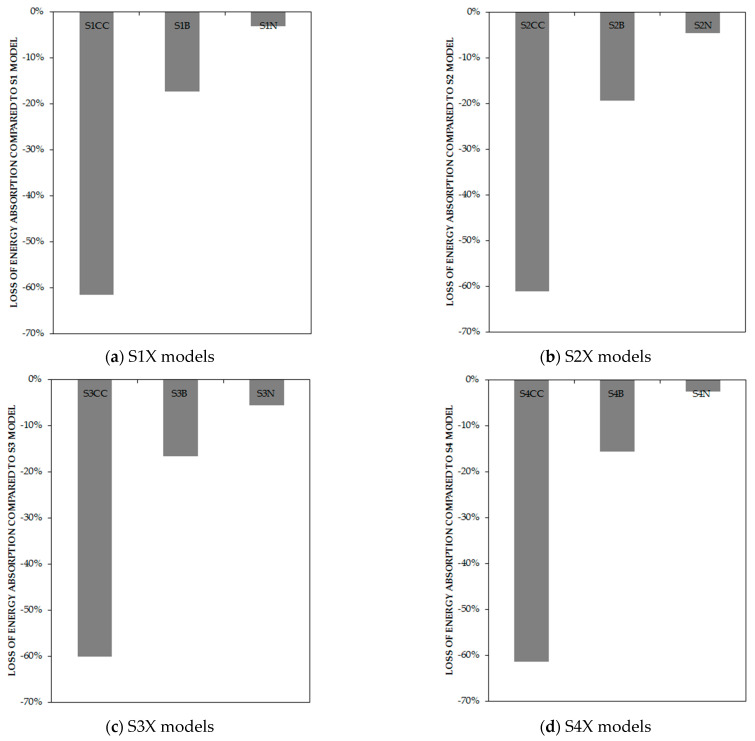
Loss of energy absorption of models based on the type of connections in comparison with full-connection models by percentage.

As expected from the results of the line graphs, the column-only-connected models experienced 54.28–57.67%, the beam-only-connected 11.18–12.64%, and those fully connected to beams and 33% to columns had a 3.23–4.60% decline in ultimate strength in comparison with the classic type of connection, which is shown in Figure 9. Figure 10 represents the connection of the infill plate effects on energy dissipation. It is determined that for the column-only curb, the beam-only connection, and the full connection to beams and 33% to columns, the energy absorption reduced by 60.16–61.48%, 15.59–16.40%, and 2.51–5.52%, respectively, in comparison with the classic type of connection.

**Figure 11:**
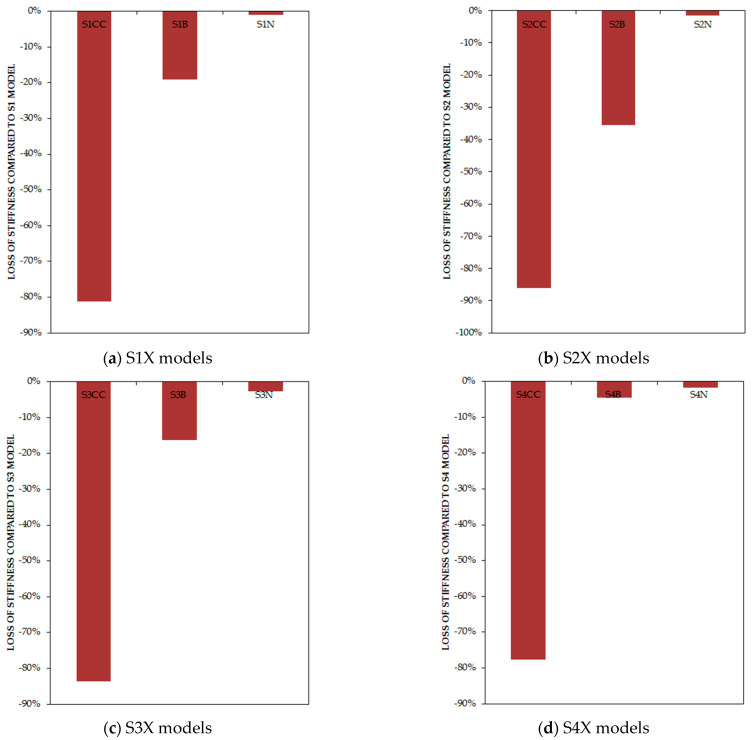
Loss of stiffness of models based on the type of connections in comparison with full-connection models by percentage.

Figure 11 represents the connection of the infill plate effects on stiffness. It is determined that for the column-only curb, the beam-only connection, and the full connection to beams and 33% to columns, the stiffness reduced by 77.61–86.15%, 4.56–35.48%, and 1.05–2.79%, respectively, in comparison with the classic type of connection.

**Figure 12:**
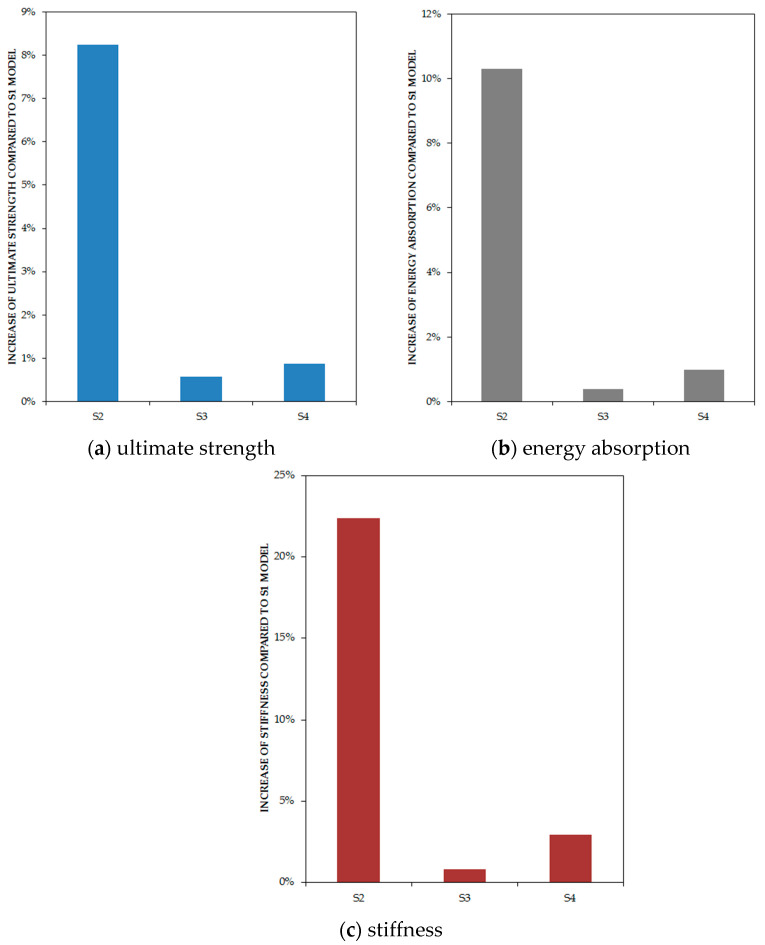
Increase in fundamental factors of FE models based on the stiffness of boundary elements in comparison with S1 model by percentage.

From the bar chart in Figure 12, it can be observed that an increase in the stiffness only in one column increased the ultimate strength to more than 8%, but this trend was below 1% for models with increased stiffness in both the first and second beams or the top beam. Increasing the stiffness of one column increased more than 10% of the energy absorption, and increasing the stiffness of the first and second floors’ beams or the top beam increased less than 1% of the energy absorption.

## 4. Conclusions

Based on the extracted result, it can be observed that the classic type of connection between infill plate and boundary elements (full connection) had the desirable performance; however, the aforementioned type could increase the demands on the columns. Accordingly, designers prefer to detach the connection of the infill plate to columns to achieve an economical design of columns. It is noteworthy to mention that according to the result, SPSWs with only beam connection can achieve considerably lower values in comparison with the classic type of SPSWs. Only 33% of the connection of the infill plate to the columns to some extent can eliminate the negative result of this type of connection. In addition, this connection has been expanded from the center of the column, which can keep the stress far from the beam-to-column-connection region. On average, the reductions in ultimate strength for the column-connected, for the beam-only-connected, and partially connected columns were 56%, 12.16%, and 4%, respectively. The decreases in energy absorption were 61%, 17%, and 3%, respectively. Furthermore, the result shows that the performance of the columns-only connection is not reliable. Even though the beams-only connection also had a considerable loss in fundamental factors, this can be rectified to some extent by the 33% connection of the infill plate to columns. Regarding the increase in stiffness in the boundary elements, it can be mentioned that although increasing the stiffness of the top beam had a more positive response in comparison with the first and second floors’ beams, this amount was negligible and the effect of the increase in the stiffness of one column had a much more positive result in comparison with the changes.

## Figures and Tables

**Table 1:** Models and changes in boundary elements.

Model	Infill Plate’s Connection to	100% Increase in Stiffness of
**S1**	COLUMN AND BEAM	-
**S1CC**	COLUMN	-
**S1B**	BEAM	-
**S1N**	BEAM AND 33% OF COLUMN	-
**S2**	COLUMN AND BEAM	ONE OF THE COLUMNS
**S2CC**	COLUMN	ONE OF THE COLUMNS
**S2B**	BEAM	ONE OF THE COLUMNS
**S2N**	BEAM AND 33% OF COLUMN	ONE OF THE COLUMNS
**S3**	COLUMN AND BEAM	FIRST AND SECOND BEAMS
**S3CC**	COLUMN	FIRST AND SECOND BEAMS
**S3B**	BEAM	FIRST AND SECOND BEAMS
**S3N**	BEAM AND 33% OF COLUMN	FIRST AND SECOND BEAMS
**S4**	COLUMN AND BEAM	TOP BEAM
**S4CC**	COLUMN	TOP BEAM
**S4B**	BEAM	TOP BEAM
**S4N**	BEAM AND 33% OF COLUMN	TOP BEAM

**Table 2:** Results of models.

Model	Ultimate Strength (kN)	Energy Absorption (kN.mm)	Stiffness (kN/mm)
**S1**	1517.48	159,684.31	112.58
**S1CC**	651.383	61,517.52	21.25
**S1B**	1328.06	131,938.53	91.03
**S1N**	1468.47	154,808.8	111.4
**S2**	1642.51	176,135.82	137.77
**S2CC**	750.877	68,435.77	19.08
**S2B**	1410	141,959.05	88.89
**S2N**	1578.43	168,138.92	135.51
**S3**	1526.32	160,315.21	110.5
**S3CC**	685.167	63,875.76	18.15
**S3B**	1333.33	133,752.2	92.38
**S3N**	1459.46	151,460.3	108.28
**S4**	1530.86	161,259.91	115.91
**S4CC**	648	62,257.4	25.95
**S4B**	1359.68	136,123.05	110.62
**S4N**	1460.47	157,209.73	113.91
